# Oxytocin and vasopressin increase male-directed threats and vocalizations in female macaques

**DOI:** 10.1038/s41598-018-36332-0

**Published:** 2018-12-20

**Authors:** Yaoguang Jiang, Michael L. Platt

**Affiliations:** 1 Department of Neuroscience, Perelman School of Medicine, University of Pennsylvania, Philadelphia, PA 19104 USA; 2 Department of Psychology, School of Arts and Sciences, University of Pennsylvania, Philadelphia, PA 19104 USA; 3 Marketing Department, the Wharton School, University of Pennsylvania, Philadelphia, PA 19104 USA

## Abstract

In a previous study, we reported that intranasal delivery of both oxytocin (OT) and arginine vasopressin (AVP) to male macaques relaxes spontaneous social interactions, flattens the existing dominance hierarchy, and increases behavioral synchrony with other monkeys. Here we report that intranasal OT and AVP administration modulates the behaviors of female macaque monkeys, but in robustly different ways from males. Most notably, both neuropeptides increase threatening and vocalization behaviors of females when they encounter males, and these behaviors effectively increase the social status of females over males. While OT and AVP heighten the confrontational nature of intersexual encounters, both peptides relax interactions between females. Finally, as previously reported for males, treating an individual female monkey with OT or AVP significantly modulates the behavior of her non-treated partner. Together, these findings show that OT and AVP can either inhibit or promote aggression, depending on sex and behavioral context, and call for a more careful, systematic examination of the functions of these neuropeptides in both sexes, especially in the context of therapeutics for human social disorders.

## Introduction

The neuropeptides oxytocin (OT) and arginine vasopressin (AVP) contribute to mammalian reproductive behaviors, including mating^1,2^, pair-bonding^3–7^, lactation^8–11^, and mother-infant bonding^12–15^. More recently, both neuropeptides have been linked to other aspects of mammalian social behavior including affiliation, communication, and social cognition^16–22^. For example, a single intranasal dose of OT in healthy humans increases trust^23^, generosity^24^, and empathy^25^. Although OT and AVP are often referred to as ‘prosocial’ neuropeptides, their effects are not consistently positive. OT is reported to increase negative social judgments^26^, heighten out-group bias^27^, and amplify anxiety to unpredictable threats^28^. In addition, high plasma levels of OT and AVP have been associated with depression^29–33^ as well as anxiety disorders^34–36^.

Further complicating the matter, both OT and AVP systems are sexually dimorphic^37^. In a number of rodent species, AVP innervation in the brain and AVP plasma concentration are higher in males than females^37–39^. In humans, preadolescent and adolescent boys^40^ as well as adult men^30,41,42^ show higher plasma concentrations of AVP compared with women. By contrast, work in animals^43^ and humans^44^ has found higher plasma and central concentrations of OT in females than males. There are also sex-specific differences in the distribution of centrally expressed OT and AVP receptors, namely OTR and V1aR^37,45–47^.

This sexual dimorphism in neuroanatomy underlies various sex-specific behaviors. For example, AVP plays a stronger role in social recognition in male rats than in females^48^, and it also shapes partner preferences in male but not female prairie voles^49,50^. By contrast, OT more strongly affects partner preference in female prairie voles than in males^51,52^. Despite extensive research in rodents, however, few studies have directly compared how OT and AVP may shape male and female behavior differently in either humans or nonhuman primates. Understanding the potential interaction between sex and neuropeptide function in primates is a high priority, since both OT and AVP are implicated in the etiology of disorders such as schizophrenia^53^, autism^54–58^, depression^29–33^, and borderline personality disorder^59^, all of which show sex biases in prevalence, symptom severity, and treatment responses^60–62^. For example, autism is 3–5 times more likely in males^63,64^. By contrast, women are diagnosed with depression twice as often as men are^65,66^. Thus, better understanding of sex differences in neuropeptide systems in health and disease may provide insights into new, more personalized treatments^67–69^.

To bridge the gap between rodent and human studies on the interaction of sex and neuropeptide function, we examined the effects of intranasal application of aerosolized OT and AVP on spontaneous social behavior in rhesus macaques. Like humans, macaques live in large, hierarchical, mixed-sex groups^70^, engage in complex social interactions^71,72^, and largely use visual and vocal signals to communicate^73^. These behaviors are mediated by a network of cortical and subcortical brain areas that appear to be homologous with the human social brain network^74–76^. Together these factors make macaque monkeys the ideal animal model for studying the neurobiology of social cognition and social deficits associated with psychiatric disorders^77–79^. In a prior study^80^, we found intranasal treatment of male macaques with both OT and AVP relaxed social encounters, flattened the existing social hierarchy, and enhanced the temporal synchrony of reciprocal behaviors between individuals. Here we used the exact same experimental design to examine the effects of intranasal OT and AVP on the behavior of female macaques. Instead of replicating the prosocial effects observed in males, we found to our surprise that both neuropeptides produced the opposite effects in females. Most importantly, both OT and AVP increased females’ threatening and vocalization behaviors towards males but not towards other females.

## Results

### Baseline female behavior towards males and females

**Figure 1:**
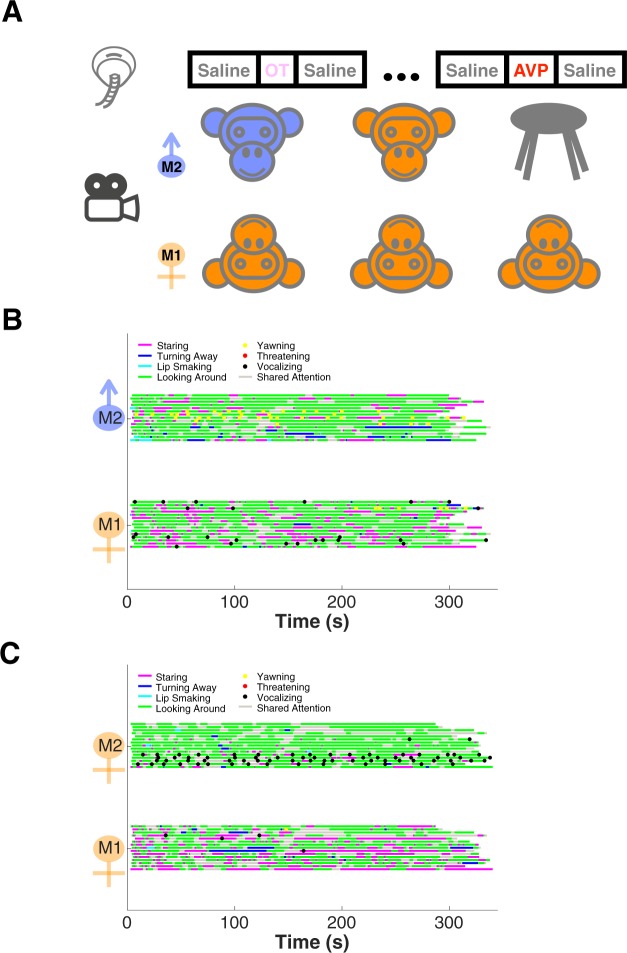
Experimental design and activity budgets. (**A**) Experimental design: one monkey (M1, female) receives saline, oxytocin (OT), or arginine vasopressin (AVP) treatments via intranasal nebulization prior to facing another monkey (M2, male or female) or an empty chair for 5 minutes in close proximity. (**B**) Example set of activity budgets from one female monkey (B) facing 3 male monkeys, each in 5 consecutive sessions (15 sessions in total). M1 inhaled saline; M2s did not. (**C**) Example set of activity budgets from the same female monkey (B) facing 3 female monkeys, each in 5 consecutive sessions (15 sessions in total). M1 inhaled saline; M2s did not.

To maximize the ecological validity and translational potential of our study, we probed the effects of OT and AVP on spontaneous, naturally-occurring social behaviors. We recorded a series of 5-minute long videos of pairs of adult macaques facing each other in close proximity. They were free to interact without danger of physical contact (see Fig. 1A, Methods as well as Supplementary Video). Prior to each session, one monkey (M1) inhaled saline (serving as a baseline for comparison), OT, or AVP via a pediatric nebulizer^81^, whereas the other monkey (M2) did not receive any treatment. M1 was always a female monkey (n = 4), whereas M2 could be either a male (n = 3), a female (n = 3), or an empty primate chair as a nonsocial control. Both monkeys’ behaviors were rated offline by 1–3 independent observers and subsequently converted to a set of activity budget plots (overall concordance across observers = 0.85, see Methods).

Fig. 1B depicts example activity budget plots from a female M1 (B) facing 3 different males in consecutive saline sessions (baseline); Fig. 1C depicts the activity budgets from the same female monkey (B) facing 3 females in consecutive saline sessions (baseline). It is clear that under saline the female’s interactions with males naturally differed from her interactions with females. Most noticeably, this female stared less at males than females (define ed as fixating directly on the other monkey, staring is considered a sign of dominance) (F-M = 35.12 ± 5.49 s; F-F = 62.37 ± 9.33 s; P = 0.023, Wilcoxon rank sum test) and vocalized more often in the presence of males than females (‘cooing’, a.k.a clear call) (F-M = 1.67 ± 0.59; F-F = 0.27 ± 0.15; P = 0.075, Wilcoxon rank sum test).

**Figure 2:**
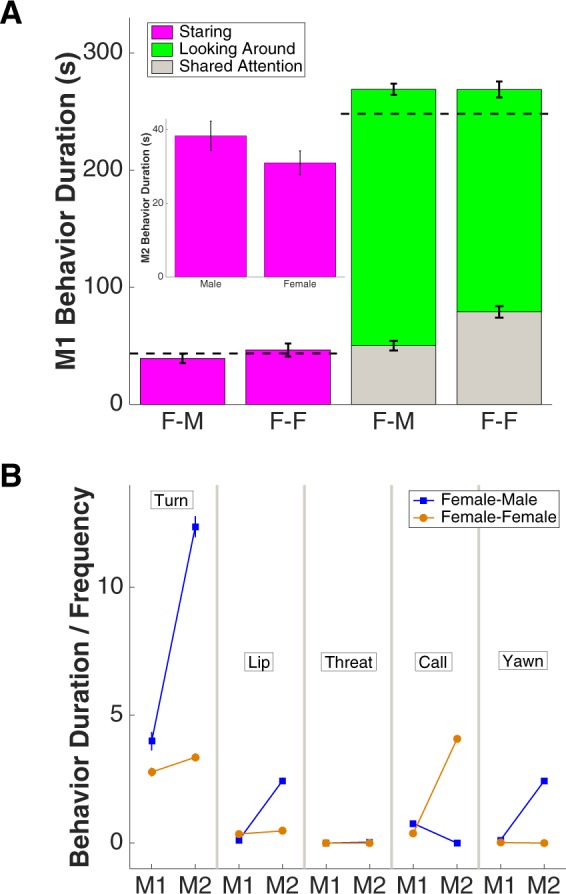
The difference between female-male and female-female interactions at baseline (under saline). (**A**) Summary of female M1s’ behaviors when facing males (i.e. F-M pairs) and females (i.e. F-F pairs). Shared attention indicates part of looking around behavior where both monkeys’ gaze is directed at the same point in space. Error bars: mean ± SEM. Insert: Summary of male and female M2s’ staring behaviors. Error bars: mean ± SEM. (**B**) Summary of other quantifiable behaviors of M1s and M2s. X axis, left to right: turning away, lip smacking, threatening, calling, yawning behavior. Error bars: mean ± SEM.

At the population level (n = 60 female-male and 60 female-female interactions under saline), there was no significant difference in female M1s’ staring behavior when facing male or female M2s (F-M = 39.23 ± 3.95 s; F-F = 46.48 ± 5.54 s; P = 0.193, Wilcoxon rank sum test) (Fig. 2A, left), but female-male pairs looked at the same objects less often than female-female pairs (shared attention, F-M = 50.21 ± 4.14 s; F-F = 78.99 ± 4.87 s; P = 0.000, Wilcoxon rank sum test) (Fig. 2A, right). There was also no significant difference between male and female M2s’ staring (male = 38.37 ± 3.95 s; female = 31.03 ± 3.23 s; P = 0.137, Wilcoxon rank sum test) (Fig. 2A, insert). By contrast, other behaviors of male and female M2s differed more dramatically from each other. Specifically, compared with female M2s, male M2s turned away more (sign of subordination, male = 12.38 ± 0.40 s; female = 3.36 ± 0.14 s; P = 0.000, Wilcoxon rank sum test), lip smacked more (sign of affiliation, male = 2.44 ± 0.77 s; female = 0.47 ± 0.22 s; P = 0.001, Wilcoxon rank sum test), and yawned more (associated with dominance, male = 2.43 ± 0.52; female = 0; P = 0.000, Wilcoxon rank sum test). By contrast, female M2s vocalized more when compared with males (male = 0; female = 4.07 ± 0.87; P = 0.000, Wilcoxon rank sum test) (Fig. 2B).

In summary, female-male interactions naturally differed from female-female interactions. In general, males were more alert and active during their interactions with females, which could be observed through both dominating and affiliative behaviors. Furthermore, females never threatened (and rarely yawned) whereas males never vocalized under these conditions.

### Intranasal OT and AVP increase female aggression towards males but not females

**Figure 3:**
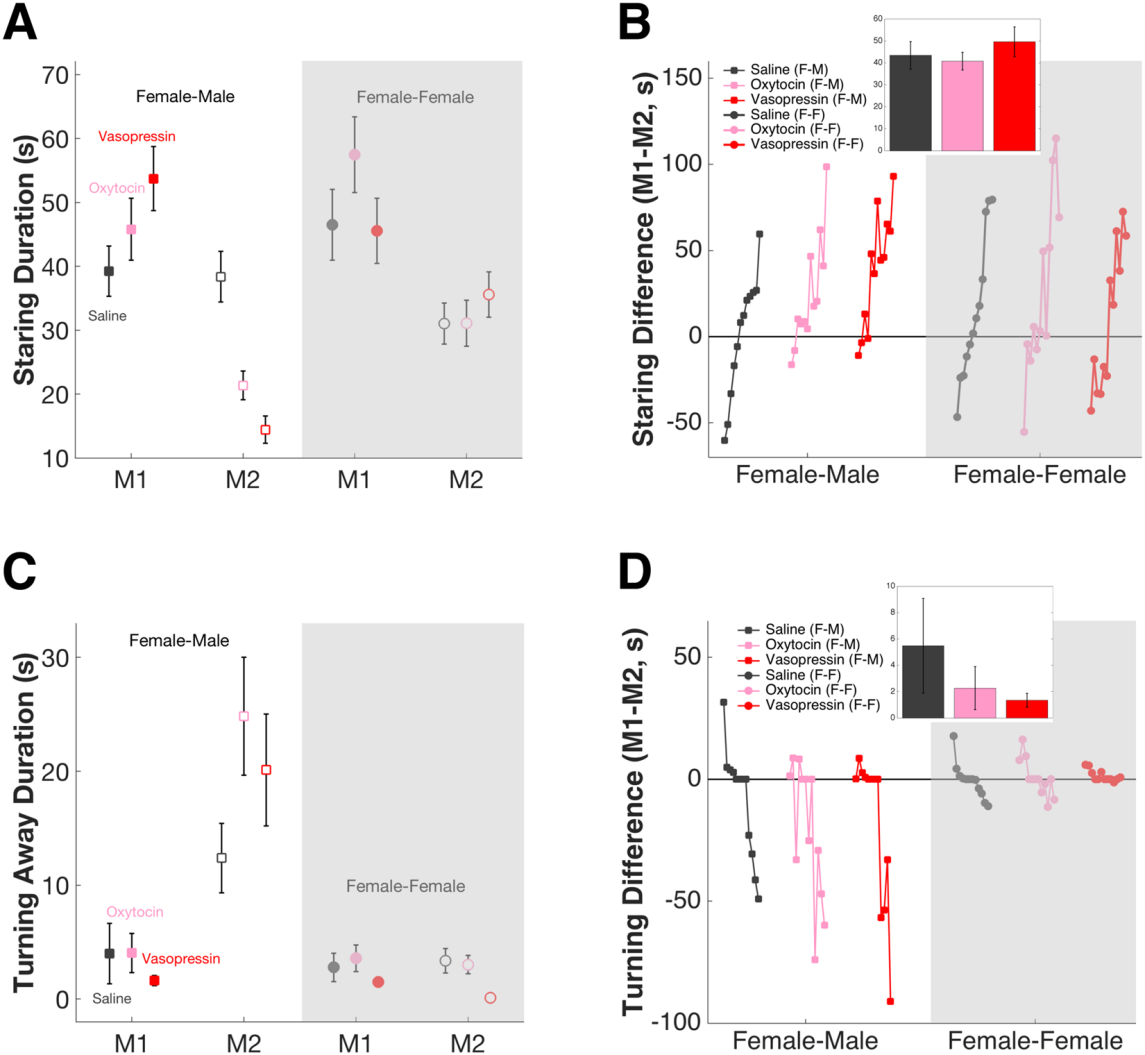
OT and AVP promote dominance gain of females over males. (**A**) Overall, OT and AVP reduce staring by male M2s. X axis, left: F-M pair; right: F-F pair. Error bars: mean ± SEM. (**B**) OT and AVP increase the relative dominance of females over males (left, F-M pairs) but not other females (right, F-F pairs), as measured by the difference between M1 and M2 staring durations. Each data point on each curve represents one monkey pair; the pairs are ordered according to staring difference under saline (grey lines); greater staring difference corresponds to higher dominance of M1 over M2. Insert: Compared with saline (grey), OT (pink) or AVP (red) does not change M1’s staring at an empty chair. Y axis: M1 staring durations (s); error bars: mean ± SEM. (**C**) OT and AVP tend to increase turning away by male M2s while decreasing turning away by female M2s. X axis, left: F-M pair; right: F-F pair. Error bars: mean ± SEM. (**D**) OT and AVP increase the relative dominance of females over males (left, F-M pairs) but not other females (right, F-F pairs), as measured by the difference between M1 and M2 turning away durations. Each data point on each curve represents one monkey pair; the pairs are ordered according to turning difference under saline (grey lines); greater turning difference corresponds to lower dominance of M1 over M2. Insert: Compared with saline (grey), OT (pink) or AVP (red) does not change M1’s turning away from an empty chair. Y axis: M1 turning away durations (s); error bars: mean ± SEM.

For the population (n = 120 saline, 120 OT, 120 AVP sessions), delivering OT or AVP intranasally (25 IU delivered in 1 ml saline vehicle) to female M1s did not significantly alter their staring behavior regardless of M2 sex (F-M saline = 39.23 ± 3.95 s, OT = 45.78 ± 4.83 s, AVP = 53.71 ± 4.99 s; F-F saline = 46.48 ± 5.54 s, OT = 57.47 ± 5.92 s, AVP = 45.53 ± 5.09 s; 2-way ANOVA, effect of sex F (1, 354) = 0.74, P = 0.389; effect of treatment F (2, 354) = 1.63, P = 0.197; interaction F (2, 354) = 2.10, P = 0.124). However, delivering both OT and AVP to female M1s significantly altered staring back by untreated M2s (male saline = 38.37 ± 3.95 s; OT = 21.36 ± 2.23 s, AVP = 14.42 ± 2.13 s; female saline = 31.03 ± 3.23 s, OT = 31.08 ± 3.59 s, AVP = 35.57 ± 3.53 s; 2-way ANOVA, effect of sex F (1, 354) = 9.11, P = 0.003; effect of treatment F (2, 354) = 5.50, P = 0.004; interaction F (2, 354) = 10.13, P = 0.000). Specifically, both OT and AVP inhalation by female M1s drastically reduced staring by male M2s (1-way ANOVA F (2, 177) = 18.15, P = 0.000; multiple comparison saline vs OT P = 0.000, saline vs AVP P = 0.000) but not female M2s (1-way ANOVA F (2, 177) = 0.57, P = 0.566) (Fig. 3A). Because direct staring is an expression of dominance, OT and AVP appeared to increase female dominance over male M2s (reflected in a positive shift in M1-M2 staring difference, saline = −116.36 to 82.54 s, OT = −39.50 to 168.05 s, AVP = −56.02 to 184.96 s; 1-way ANOVA F (2, 177) = 12.21, P = 0.000) (Fig. 3B, left). By contrast, the dominance order among females remained largely unchanged (M1-M2 staring difference, saline = −71.61 to 185.60 s, OT = −89.28 to 140.27 s, AVP = −101.72 to 140.51 s; 1-way ANOVA F (2, 177) = 1.53, P = 0.221) (Fig. 3B, right). Importantly, the average time M1 spent staring at an empty chair was the same under all conditions (saline = 43.45 ± 6.30 s; OT = 40.78 ± 4.03 s; AVP = 49.61 ± 6.80 s; 1-way ANOVA F (2, 57) = 0.60, P = 0.551) (Fig. 3B, insert), indicating that OT and AVP did not alter motor functions including the scanning patterns of monkeys.

Next we examined the effects of neuropeptide treatments on turning away behaviors. Again we found neither neuropeptide altered turning away by M1 regardless of M2 sex (F-M saline = 3.98 ± 2.65 s, OT = 4.04 ± 1.73 s, AVP = 1.61 ± 0.43 s; F-F saline = 2.78 ± 1.24 s, OT = 3.57 ± 1.17 s, AVP = 1.48 ± 0.41 s; 2-way ANOVA with bootstrapping, effect of sex F (1, 354) = 0.24, P = 0.545; effect of treatment F (2, 354) = 1.30, P = 0.267; interaction F (2, 354) = 0.07, P = 0.878). Even though OT and AVP treatment did not significantly altered overall turning away by untreated M2s (male saline = 12.37 ± 3.06 s; OT = 24.84 ± 5.18 s, AVP = 20.11 ± 4.93 s; female saline = 3.36 ± 1.09 s, OT = 3.02 ± 0.82 s, AVP = 0.10 ± 0.10 s; 2-way ANOVA with bootstrapping, effect of sex F (1, 354) = 41.49, P = 0.000; effect of treatment F (2, 354) = 1.81, P = 0.143; interaction F (2, 354) = 2.31, P = 0.076), both neuropeptides had a tendency to increase turning away by untreated male M2s (Kruskal-Wallies test Chi-square = 4.62, P = 0.099) (Fig. 3C, left). By contrast, AVP but not OT significantly decreased turning away by untreated female M2s (Kruskal-Wallies test Chi-square = 17.26, P = 0.000; multiple comparison saline vs OT P = 0.988, saline vs AVP P = 0.001) (Fig. 3C, right). Because turning away is a sign of subordination, both OT and AVP in effect increased female dominance over males (reflected in a negative shift in M1-M2 turning difference, saline = −103.85 to 158.03 s, OT = −150.78 to 38.11 s, AVP = −151.25 to 16.49 s; Kruskal-Wallies test Chi-square = 5.54, P = 0.063) (Fig. 3D, left), whereas the dominance order among females remained unchanged (saline = −48.67 to 46.16 s, OT = −28.31 to 45.12 s, AVP = −6.04 to 16.30 s; Kruskal-Wallies test Chi-square = 1.07, P = 0.586) (Fig. 3D, right). Importantly, the average time M1 spent turning away from an empty chair was the same under all conditions (saline = 5.48 ± 3.11 s; OT = 2.27 ± 1.41 s; AVP = 1.35 ± 0.46 s; Kruskal-Wallies test Chi-square = 2.12, P = 0.346) (Fig. 3D, insert).

To briefly sum up, OT and AVP both altered female-male interactions more substantially than female-female interactions. In terms of both staring (dominant) and turning (subordinate) behaviors, the most robust modulation was observed in the untreated M2s instead of the treated M1s. The overall effect of OT and AVP was a relative gain in dominance status for females when they encountered males, but not when they faced other females.

**Figure 4:**
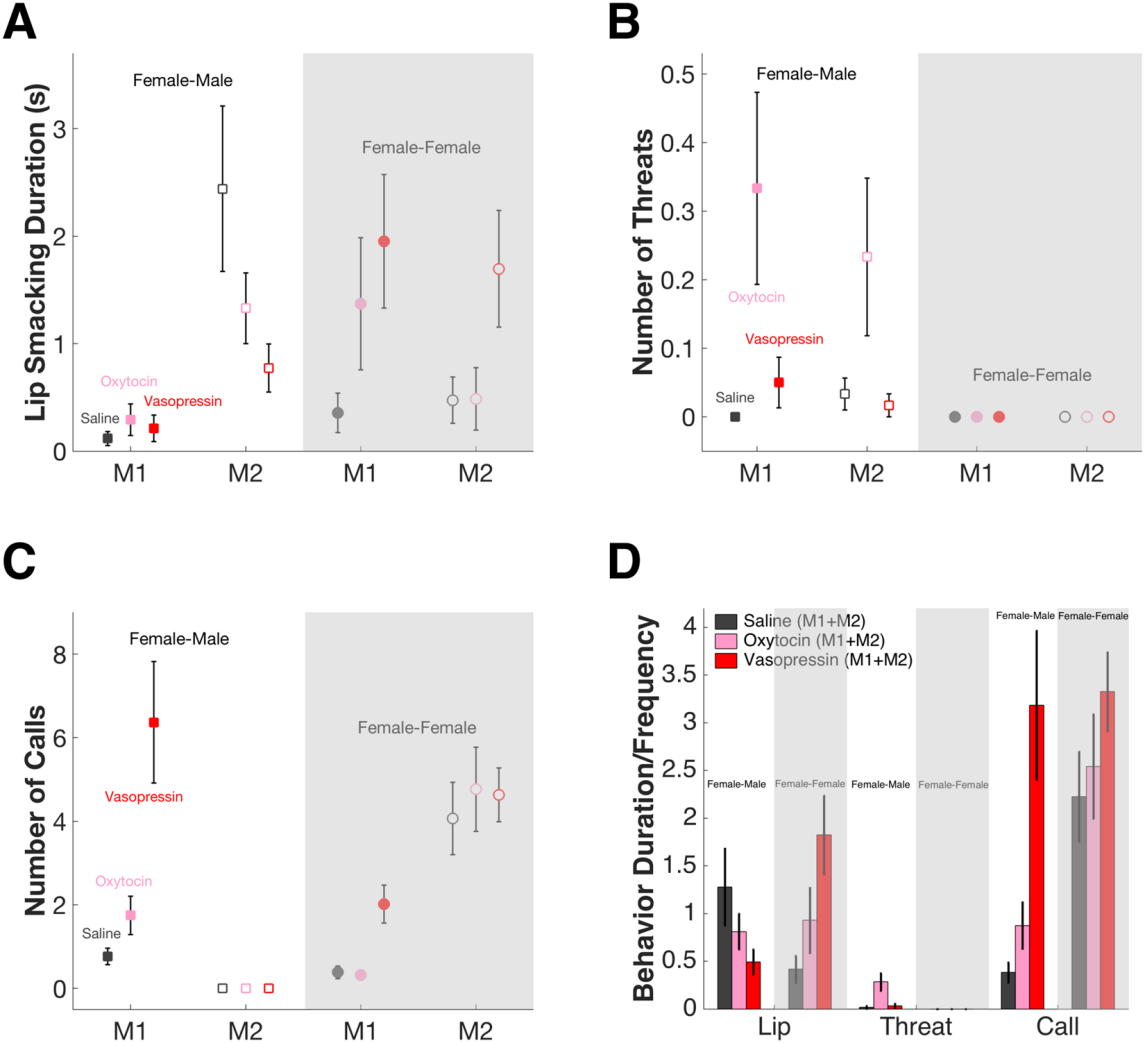
OT and AVP promote confrontational behavior during female-male interactions but affiliative behaviors during female-female interactions. (**A**) OT and AVP decrease lip smacking from male M2s but increase lip smacking within F-F pairs. X axis, left: F-M pair; right: F-F pair. Error bars: mean ± SEM. (**B**) OT and AVP increase threats within F-M pairs but not F-F pairs. X axis, left: F-M pair; right: F-F pair. Error bars: mean ± SEM. (**C**) OT and AVP increase calling by female M1s. X axis, left: F-M pair; right: F-F pair. Error bars: mean ± SEM. (**D**) Summary of M1 plus M2 behavioral changes under OT and AVP. X axis, left to right: lip smacking, threatening, calling behavior. Unshaded areas: F-M pair; shaded areas: F-F pairs. Error bars: mean ± SEM.

We next investigated how OT and AVP inhalation by female M1s induced changes in the behavior of their male partners, since there was no change in staring or turning away by female M1s themselves. We found both OT and AVP modulated lip smacking by female M1s differently depending on the sex of her partner. Specifically, M1 lip smacked more when facing female instead of male M2s (F-M saline = 0.12 ± 0.07 s, OT = 0.29 ± 0.15 s, AVP = 0.21 ± 0.12 s; F-F saline = 0.36 ± 0.18 s, OT = 1.37 ± 0.61 s, AVP = 1.95 ± 0.62 s; 2-way ANOVA, effect of sex F (1, 354) = 11.15, P = 0.001; effect of treatment F (2, 354) = 2.69, P = 0.069; interaction F (2, 354) = 2.02, P = 0.134). Correspondingly, lip smacking by male M2s decreased whereas lip smacking by female M2s increased following neuropeptide treatment of female M1 (male saline = 2.44 ± 0.77 s, OT = 1.33 ± 0.33 s, AVP = 0.77 ± 0.22 s; female saline = 0.47 ± 0.22 s, OT = 0.49 ± 0.29 s, AVP = 1.70 ± 0.54 s; 2-way ANOVA, effect of sex F (1, 354) = 3.03, P = 0.083; effect of treatment F (2, 354) = 0.78, P = 0.461; interaction F (2, 354) = 5.40, P = 0.005; multiple comparison male saline vs OT P = 0.259, male saline vs AVP P = 0.049, female saline vs OT P = 0.999, female saline vs AVP P = 0.057) (Fig. 4A). Importantly, monkeys never lip smacked at an empty chair under any treatment condition, indicating that the effect of OT and AVP treatment on lip smacking behavior was specifically social.

Remarkably, female M1s never threatened either male or female partners under saline, but threatened males following neuropeptide treatment, particularly OT. In contrast, there was no change in aggression towards other females after OT or AVP treatment (F-M saline = 0, OT = 0.33 ± 0.14, AVP = 0.05 ± 0.04; F-F saline = OT = AVP = 0; 2-way ANOVA, effect of sex F (1, 354) = 7.01, P = 0.009; effect of treatment F (2, 354) = 4.63, P = 0.010; interaction F (2, 354) = 4.63, P = 0.010; multiple comparison F-M saline vs OT P = 0.013, F-M saline vs AVP P = 0.227). Correspondingly, male M2s were more likely to threaten female M1s treated with OT, whereas female M2s did not threaten in any condition (male saline = 0.03 ± 0.02, OT = 0.23 ± 0.11, AVP = 0.02 ± 0.02; female saline = OT = AVP = 0; 2-way ANOVA, effect of sex F (1, 354) = 5.72, P = 0.017; effect of treatment F (2, 354) = 3.11, P = 0.046; interaction F (2, 354) = 3.11, P = 0.046; multiple comparison male saline vs OT P = 0.097, male saline vs AVP P = 0.984) (Fig. 4B). Importantly, monkeys never threatened at an empty chair under any treatment condition.

Finally, both OT and AVP increased vocalizations by female M1s (F-M saline = 0.77 ± 0.20, OT = 1.75 ± 0.46, AVP = 6.37 ± 1.45; F-F saline = 0.38 ± 0.15, OT = 0.32 ± 0.10, AVP = 2.02 ± 0.46; 2-way ANOVA, effect of sex F (1, 354) = 14.63, P = 0.000; effect of treatment F (2, 354) = 17.90, P = 0.000; interaction F (2, 354) = 4.88, P = 0.008) but did not increase vocalizations by male or female M2s (male saline = OT = AVP = 0; female saline = 4.07 ± 0.87, OT = 4.77 ± 1.01, AVP = 4.63 ± 0.64; 2-way ANOVA, effect of sex F (1, 354) = 82.96, P = 0.000; effect of treatment F (2, 354) = 0.19, P = 0.827; interaction F (2, 354) = 0.19, P = 0.827) (Fig. 4C). Importantly, M1’s vocalization frequency was the same under all conditions when facing an empty chair (saline = 2.90 ± 1.09; OT = 0.90 ± 0.25 s; AVP = 3.10 ± 0.54 s; 1-way ANOVA F (2, 57) = 0.96, P = 0.39).

In summary, we found that OT and AVP treatments in female monkeys increased the frequency of threats and vocalizations when they confronted male conspecifics, which were returned by males with an increase in dominant behavior (threat) and a decrease in affiliative behavior (lip smack). By contrast, interactions between females became more amicable following neuropeptide treatment, reflected by an increase in affiliative behavior (lip smack) (Fig. 4D).

### Intranasal OT and AVP amplify intrasexual and intersexual behaviors

**Figure 5:**
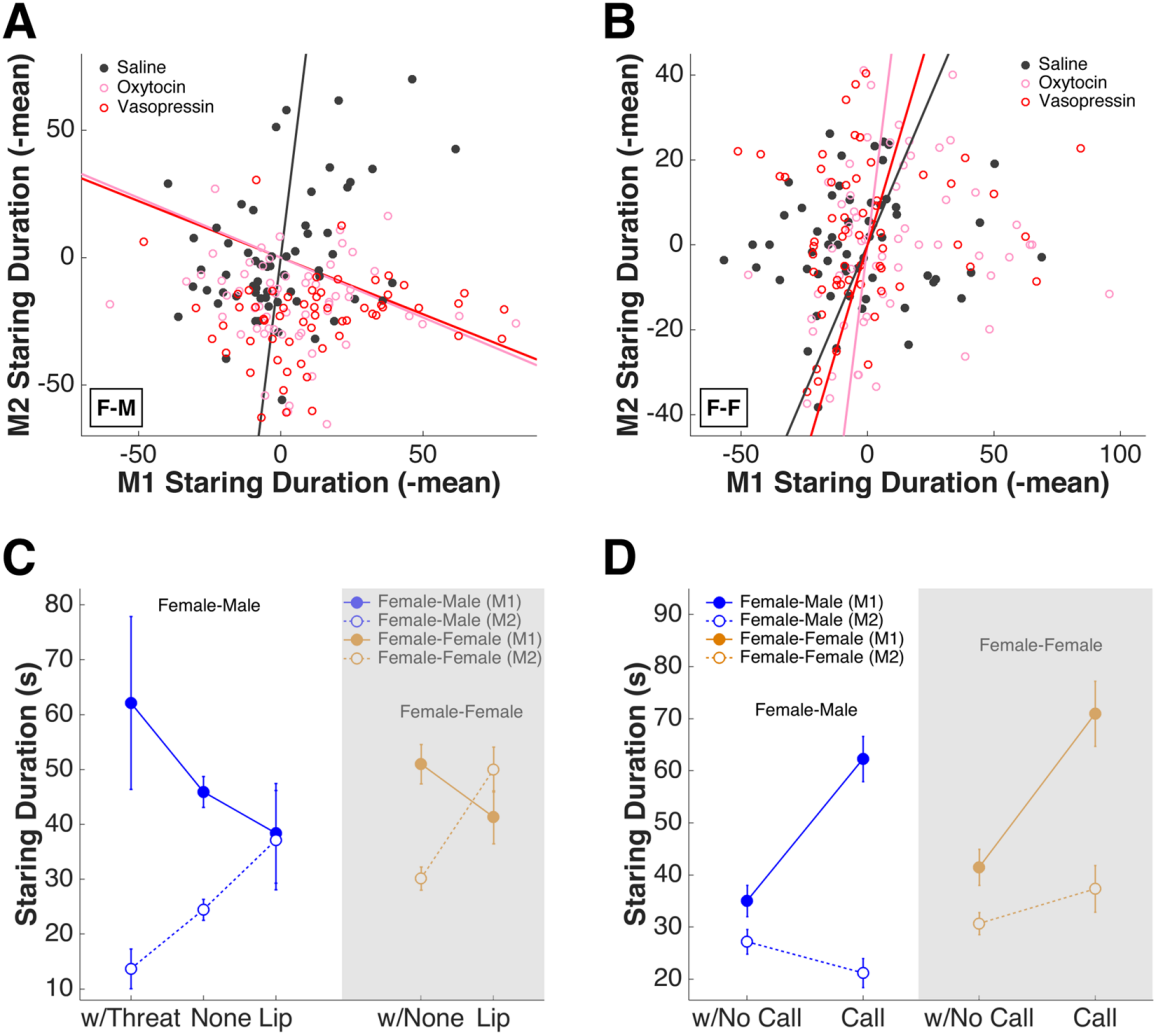
The correlations among different behaviors. (**A**) In female-male pairs, under OT and AVP female M1s’ staring increases as male M2s’ staring decreases. Straight lines indicate linear regression fits for saline (grey), OT (pink), and AVP (red) data. (**B**) Such correlation does not exist in female-female interactions. Straight lines indicate linear regression fits for saline (grey), OT (pink), and AVP (red) data. (**C**) Compared to baseline, M1 making threats is linked to more prolonged staring from M1 and reduced staring from M2. In contrast, M1 lip smacking is linked to reduced staring from M1 and increased staring from M2. This is true for both F-M (left) and F-F (right) pairs. X axis: w/Threat: sessions during which M1 made threats; None: sessions during which M1 made neither threats nor lip smacks; Lip: sessions during which M1 made lip smacks. Error bars: mean ± SEM. (**D**) In F-M pairs (left), M1 making cooing calls is linked to more prolonged staring from M1 and reduced staring from M2. In F-F (right) pairs, increase in M1 calling is linked to increase in M1 staring as well as a smaller increase in M2 staring. X axis: w/No Call: sessions during which M1 did not make any calls; Call: sessions during which M1 made calls. Error bars: mean ± SEM.

We next aimed to clarify the sequence of neuropeptide effects on behavior. First, we noted that during female-male interactions, staring by female M1s increased slightly following OT and AVP (1-way ANOVA, F (2, 177) = 2.47, P = 0.088) (Fig. 3A, left). This increase in female M1 staring was correlated with a decrease in staring by male M2s, suggesting females asserted more dominance over males following OT and AVP treatments (saline r = 0.36, P = 0.005, OT/AVP (minus saline baseline) r = −0.16, P = 0.076) (Fig. 5A). By contrast, no such correlation was present during interactions amongst females (saline r = 0.11, P = 0.417, OT/AVP (minus saline baseline) r = 0.15, P = 0.105) (Fig. 5B).

OT and AVP also caused female M1s to threaten males more frequently (Fig. 4B). By contrast, OT and AVP caused female M1s to express more affiliative gestures (i.e. lip smacking) towards other females (Fig. 4A). Threats from female M1s were correlated with more staring by M1 and less staring by M2 (Fig. 5C, w/Threat). On the other hand, lip smacks from female M1s were correlated with less staring from M1 and more staring from M2 (Fig. 5C, Lip). These effects were true for both female-female and female-male interactions (F-M: M1 w/Threat = 62.15 ± 15.73 s, M1 None = 45.93 ± 2.79 s, M1 Lip = 38.39 ± 9.08 s; M2 w/Threat = 13.69 ± 3.65 s, M2 None = 24.46 ± 1.89 s, M2 Lip = 37.15 ± 9.07 s; 2-way ANOVA, effect of M1 behavior F (2, 358) = 0.14, P = 0.870; effect of M1vsM2 F (1, 358) = 13.54, P = 0.000; interaction F (2, 358) = 3.08, P = 0.047) (F-F: M1 w/None = 51.01 ± 3.58 s, M1 Lip = 41.34 ± 4.82 s; M2 w/None = 30.14 ± 2.13 s, M2 Lip = 50.00 ± 4.09 s; 2-way ANOVA, effect of M1 behavior F (1, 356) = 0.80, P = 0.373; effect of M1vsM2 F (1, 356) = 1.15, P = 0.285; interaction F (1, 356) = 6.69, P = 0.010). Thus, OT and AVP treatment shaped visual communication between females and other macaques in sex-specific fashion.

Finally, we noticed that female macaques showed sex-specific vocalizations, and this behavior also was modulated by OT and AVP. Indeed, we found vocalizations by female M1s were correlated with more staring by M1s and less staring back by male M2s (F-M: M1 no call = 35.03 ± 2.98 s, M1 call = 62.29 ± 4.33 s; M2 no call = 27.18 ± 2.39 s, M2 call = 21.18 ± 2.79 s; 2-way ANOVA, effect of M1 behavior F (1, 356) = 11.47, P = 0.001; effect of M1vsM2 F (1, 356) = 60.88, P = 0.000; interaction F (1, 356) = 28.10, P = 0.000). By contrast, vocalizations by female M1s did not decrease staring by female M2s (F-F: M1 no call = 41.47 ± 3.46 s, M1 call = 70.98 ± 6.26 s; M2 no call = 30.68 ± 2.12 s, M2 call = 37.33 ± 4.49 s; 2-way ANOVA, effect of M1 behavior F (1, 356) = 20.08, P = 0.000; effect of M1vsM2 F (1, 356) = 30.31, P = 0.000; interaction F (1, 356) = 8.02, P = 0.005) (Fig. 5D). These results indicate that although females vocalized towards both males and females, males and females reacted to these vocalizations in different ways.

**Figure 6:**
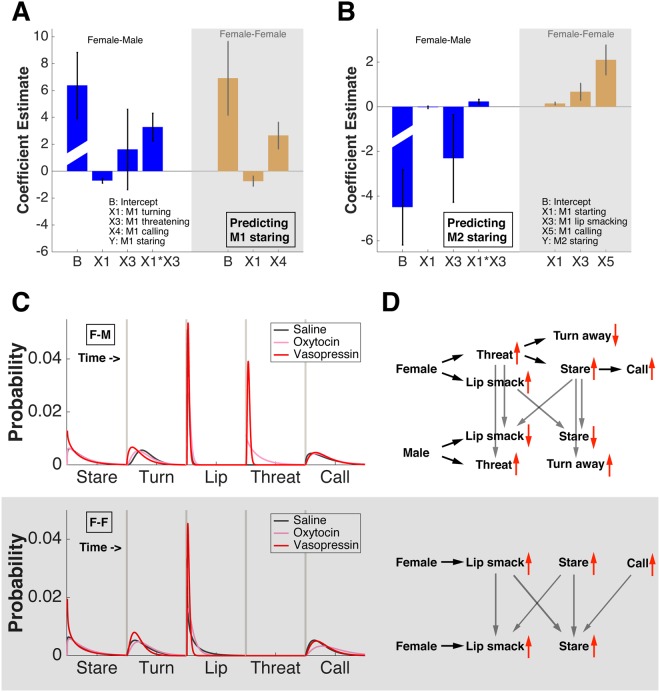
The sequence of behavioral events and how they are modulated by neuropeptides during female-male and female-female interactions. (**A**) General linear models predicting female M1’s staring behavior during female-male (left) and female-female (right) interactions. Error bars: estimated coefficient ± SEM. (**B**) General linear models to predict M2’s staring behavior during female-male (left) and female-female (right) interactions. Error bars: estimated coefficient ± SEM. (**C**) Reaction time distributions (gamma fitted) for all significant behaviors during female-male (top) and female-female (bottom) interactions. (**D**) Proposed behavioral models to explain how OT and AVP affected intersexual and intrasexual interactions differently. Up red arrow: increase in behavior duration/frequency; down red arrow: decrease in behavior duration/frequency. Black arrow: order of behavioral events within a monkey; grey arrow: order of behavioral events across monkeys.

To determine how neuropeptides influence the sequence of behaviors evoked during an interaction, we entered all significant behavioral variables into general linear models (GLMs) and found that different models accounted for female-male interactions and female-female interactions. During interactions between females and males, both turning away and threatening (in the absence of turning) predicted how staring by female M1s was modulated by neuropeptides (estimated coefficient for turn = −0.70 ± 0.18, P = 0.000; threat = 1.62 ± 2.99, P = 0.589; turn*threat = 3.27 ± 1.03, P = 0.002). By contrast, during interactions between females, turning away and calling predicted changes in staring by female M1s (estimated coefficient for turn = −0.74 ± 0.37, P = 0.050; call = −2.65 ± 0.99, P = 0.008) (Fig. 6A). We also found that both lip smacking and staring (in the absence of lip smacking) by M1 predicted changes in staring by male M2s (estimated coefficient for stare = −0.02 ± 0.05, P = 0.711; lip smack = −2.31 ± 1.97, P = 0.143; stare*lip smack = 0.23 ± 0.11, P = 0.034). By contrast, staring, lip smacking and calling by M1 predicted changes in staring by female M2s (estimated coefficient for stare = 0.15 ± 0.06, P = 0.016; lip smack = 0.67 ± 0.38, P = 0.081, call = 2.10 ± 0.66, P = 0.002) (Fig. 6B).

These and other GLM results, viewed together with the reaction time distributions of different behaviors (Fig. 6C, note how lip smacking and threatening behaviors preceded staring whereas turning and calling often co-occurred with or followed staring) lead us to propose two distinct models for how the neuropeptides OT and AVP modulate intersexual and intrasexual interactions (Fig. 6D). We hypothesize that female-male interactions are much more complex and involve a greater number of behaviors. In addition, two critical behaviors displayed by female M1s, namely staring and lip smacking, have different effects on males and females. Importantly, staring by females towards males leads to less staring and lip smacking by males, whereas in female-female pairs the opposite is true. These findings demonstrate that the same female behaviors can be interpreted as aggressive or affiliative depending on the sex of the target.

## Discussion

Oxytocin and vasopressin shape social behavior in a wide array of animals, including rodents^3,4,7,12–17,82,83^, nonhuman primates^5,6,80,81,84–88^, and humans^20–23,89–92^. Both OT and AVP systems are sexually dimorphic and interact heavily with gonadal hormones throughout development and adulthood^36,37^. Abundant evidence in rodents suggests a link between OT/AVP systems and sex-specific social behaviors^47–52,93–95^. Yet very few studies have directly compared the behavioral effects of OT and AVP on male and female primates including humans (but see^96–98^). In a prior study^80^, we showed that intranasal delivery of OT or AVP to male macaques relaxes their spontaneous social interactions with other monkeys, regardless of sex, and thereby flattens the existing status hierarchy. In the current study, using exactly the same methods, we found that OT and AVP exert completely different effects on female macaques. Both neuropeptides increase aggression and vocalizations of females when they encounter males, but not when they encounter other females. This is the first demonstration that exogenous OT and AVP alter male and female behaviors, especially aggression and affiliation, in fundamentally different ways in a nonhuman primate.

Aggressive behaviors are often extremely sexually dimorphic. In many mammals, including humans, females are often less aggressive^99–101^ and sometimes more cooperative^102–106^ than males. Males are often aggressive towards both sexes to compete for a wide range of resources, whereas females are more aggressive towards males than females^99–101,107,108^, often within the context of reproduction^109–111^. This type of temporary behavioral change, termed ‘maternal aggression,’ is considered critical for defending offspring against potential threats, especially infanticidal males^109–112^. OT has been implicated in all aspects of maternal behavior^113–116^. It specifically suppresses aggression towards one’s own offspring and increases aggression towards intruders^115,117,118^. Although AVP is known to contribute to male aggression^39^, our understanding of its role in female aggression is very limited. Our findings regarding the sex-specific effects of exogenous OT and AVP in macaque monkeys nicely correspond to recent studies in humans^97,98,119,120^ reporting neuropeptides promoting prosocial behaviors in males more than in females. It is worth noting that since exogenous OT and AVP very likely bind to each other’s receptors to modify behavior^121^, it is difficult to directly compare the effects of these two neuropeptides to each other. That being said, echoing our previous observation in male macaque monkeys^80^, here we did not observe any systematic difference in the behavioral effects of OT versus AVP treatment. We conjecture that much like in male monkeys, the effects of OT on female monkeys are also likely to be partially mediated via binding to AVP receptors in the cortex.

Although we observed an increase in intersexual aggression following neuropeptide treatment of females, we also noted an increase in affiliative behaviors between females. OT has been linked to a preference for affiliation over aggression in females (i.e. the ‘tend and befriend’ strategy^106^). This preference may help protect females against aggression by males. Increases in vocalizations following neuropeptide treatment of females may reflect attempts to recruit support of other females to protect against males^122–124^. Together, our results indicate that OT and AVP inhalation differentially affects female behavior towards males and females, possibly by recruiting different neural circuits related to reproductive behaviors. In baseline conditions, female-male and female-female interactions are readily distinguishable from each other, suggesting that rather than altering the nature of intersexual and intrasexual interactions, OT and AVP amplify pre-existing sex-specific patterns of interaction^125^.

In our previous study^80^, we reported that OT and AVP not only significantly reduce staring from both treated M1s and untreated M2s, but also shorten the latency with which dominant monkeys return stares and subordinate monkeys turn away to avert stares from other monkeys. This pattern suggested an increase in the efficiency and immediacy of communication between monkeys, consistent with an increase in social attention. In the current study, while it is somewhat surprising that neither OT nor AVP treatment significantly alters staring behavior in treated female M1s, we did find that neuropeptide treatment of female monkeys increases the frequency of threats (towards male partners) and lip smacks (towards female partners). GLMs together with reaction time analyses on different behaviors suggest that threats and lip smacks could potentially lead all other behaviors and set the tone for the subsequent interaction. Regardless of the mechanism, once OT or AVP alters the behavior of one monkey, however subtly, its effect is amplified by feedback from the untreated monkey, thus shaping the overall tenor of the social interaction. Through such chain reactions, OT and AVP regulate the nature of social interactions in profoundly sex-specific ways.

## Methods

The majority of methods and analyses used in this study were described in detail in a previous paper^80^.

### Animals

All procedures reported in this study were approved by the Institutional Animal Care and Use Committee of the University of Pennsylvania, and performed in accordance with their relevant guidelines and regulations. Four female rhesus macaques (B, C, F, Sch, 8–12 kg) and three male rhesus macaques (D, O, S, 12–16 kg) participated in the experiment. All of them had equal probabilities of being M2, but only the four females participated as M1 and received treatments. These macaques lived in a colony together (with no other monkeys) for the duration of this experiment. Cages were arranged facing toward the center of the room, along two walls, permitting all animals to be in continuous visual and auditory contact. Two of the four females (B and C) were pair-housed. All animals were between the ages of 11 and 18 at the time of the experiments and had been in the colony for at least six months. Food grabbing tests as well as caretakers/handlers’ ratings confirmed that the dominance order within this group was: O > C > B > F > S > Sch > D for the duration of the experiment. 2-way ANOVAs revealed no significant interactions between neuropeptide treatment and weight, age, or dominance order of the animals.

### Experimental Setup

In each experiment session, two monkeys faced each other face-on in an empty room, and were free to interact for 5 minutes. The monkeys sat in their respective primate chairs (Crist Instruments), and the two chairs were positioned close together without touching each other (~30 cm apart from edge to edge). This relatively unconstrained yet still well controlled setup afforded us much of the flexibility of natural social interactions without risking actual physical contact between monkeys. A video camera (Logitech, 60 fps) was positioned on the right side of M1/the left side of M2, and simultaneously recorded both monkeys’ behaviors into an MP4 file. On each day, one M1 faced six different M2s sequentially. In addition, the same M1 also faced an empty chair for 5 minutes. The order in which M1 faced the other monkeys together with the empty chair was determined randomly each day.

### Intranasal Nebulization

The procedure for intranasal OT delivery in macaque monkeys has been described in detail previously^80,81,87^. Briefly, monkeys were trained to accept a pediatric nebulizer mask (Pari Labs) over the nose and mouth. Through the nebulizer 1 ml of OT (25 IU/ml in saline; Agrilabs/Sigma Aldrich) or saline was delivered at a constant rate (0.2 ml/min) over a total of 5 minutes. Behavioral testing began 30 minutes after intranasal delivery and continued for 1–2 hours. This OT dosage and timing protocol were similar to that typically used in humans^20,89–92^ and other non-human primate studies^81,87,126,127^. We followed the same procedure for intranasal AVP delivery (25 IU in 1 ml saline; Sigma Aldrich). The same amount of neuropeptide (25 IU) was delivered to all monkeys regardless of their weights (ranging from 8–12 kg at the time of the experiment), which resulted in a dosage of ~2.1–3.1 IU/kg. Neuropeptide and saline treatments were delivered on alternating days, with each monkey receiving no more than 5 treatments per week. The same monkey never received OT and AVP treatment within the same week. The order of treatments was counterbalanced across monkeys as well as within monkeys between weeks to mitigate any possible order effects. Furthermore, to rule out the possibility that the particular sequence with which different monkey pairs were tested (after saline or neuropeptide delivery) had any impact on behavior, 1-way ANOVAs were performed on different behaviors and did not reveal any significant order effect. In addition, general linear models were constructed for different behaviors as well, with the testing order being treated as a continuous variable and measured as the estimated time passed from drug administration to behavioral testing for each pair. This analysis revealed no significant order effect either.

### Data Analysis

Each behavioral video was rated offline by 1 to 3 independent viewers, all of whom were blind to treatment conditions. Viewers used a Python GTK based, custom GUI to play and pause the video, adjust its speed, and code monkey behaviors by pressing a set of keys on the keyboard. Briefly, the ethogram used in this experiment included: (1) staring: one monkey facing forward and fixating directly on the other monkey (a sign of dominance); (2) turning away: one monkey turning at least 180° in the chair with its back facing the other monkey; (3) lip smacking: one monkey facing forward and quickly smacking its lips at the other monkey, sometimes accompanied by slight head tilt; (4) looking around: one monkey facing forward but not making direct eye contact with the other monkey; (5) yawning: one monkey opening its mouth widely and inhaling deeply; (6) threatening: one monkey staring at the other with its eyes wide open, often with open mouth and tense with the lips covering the teeth, sometimes accompanied by head jerk; (7) vocalizing: defined here as a soft, high pitch cooing, otherwise known as clear call; (8) shared attention: defined here as a portion of the looking around behavior where both monkeys’ gaze was directed away from each other, but at the same point in space.

The strings of identified behaviors and their corresponding time stamps were imported into MATLAB (Mathworks) and converted into a pair of activity budget plots via custom MATLAB scripts. When more than one viewer rated the same video (~40% of all the videos), their ratings were averaged to generate the activity budgets. For the same videos rating consistency was very high across different viewers (for example, fixation duration r = 0.66, P = 0.000; number of fixations r = 0.40, P = 0.001). The overall concordance across observers was 0.85. All subsequent data analyses were accomplished with custom MATLAB scripts.

All statistical tests were two-tailed. For hypothesis testing between two samples, a non-parametric Wilcoxon signed rank test (for paired samples) or Wilcoxon rank sum test (for un-paired samples) was used, as observed behavioral durations or frequencies often form skewed distributions with long tails^80^. For comparison among more than two samples, an ANOVA was used together with multiple comparisons (Tukey’s HSD test) when appropriate. 1-way ANOVA was replaced with Kruskal-Wallies test if the assumption of normal distribution was significantly violated. 2-way ANOVA was used in combination with a bootstrapping test to determine the corresponding P values if the assumption of normal distribution was significantly violated. Correlation coefficients were estimated with Pearson’s r. All reaction time distributions were fitted with Gamma distributions:

1 $$y=f(x|a,b)=\frac{1}{{b}^{a}{\rm{\Gamma }}(a)}{x}^{a-1}{e}^{\frac{-x}{b}}$$

where Γ is the Gamma function (${\rm{\Gamma }}(n)=(n-1)!$), a is a shape parameter, and b is a scale parameter.

## Electronic supplementary material

Supplementary figure legend

Supplementary video 1

## Data Availability

The datasets generated during and/or analyzed during the current study are available from the corresponding author upon request.
