# Supplementary material for: Oxytocin and vasopressin increase male-directed threats and vocalizations in female macaques
Source: Sci Rep. 2018 Dec 20;8:18011. doi: 10.1038/s41598-018-36332-0 (PMC6301990; doi:10.1038/s41598-018-36332-0)
Supplement: Supplementary file 1 — Supplementary figure legend [file 41598_2018_36332_MOESM1_ESM.docx]

**Title**

Oxytocin and vasopressin increase male-directed threats and vocalizations in female macaques

**Authors**

Yaoguang Jiang, Michael L. Platt

**Supplementary video**

A brief video clip (~ 1 minute) taken from one experimental session. M1 (B, female, left) inhaled OT prior to the experiment, whereas M2 (F, female, right) did not receive any treatment. A variety of behaviors can be identified from the video clip, including (not necessarily in this order) staring, lip-smacking, looking away, turning away, calling.
